# Photodynamic therapy in pediatric age: Current applications and future trends

**DOI:** 10.3389/fphar.2022.879380

**Published:** 2022-08-16

**Authors:** Luca Di Bartolomeo, Domenica Altavilla, Mario Vaccaro, Federico Vaccaro, Violetta Squadrito, Francesco Squadrito, Francesco Borgia

**Affiliations:** ^1^ Department of Clinical and Experimental Medicine, Dermatology, University of Messina, Messina, Italy; ^2^ Department of Clinical and Experimental Medicine, Pharmacology, University of Messina, Messina, Italy; ^3^ Department of Dermatology, University of Modena and Reggio Emilia, Modena, Italy; ^4^ Department of Human Pathology in Adult and Developmental Age “Gaetano Barresi, Pediatryˮ, University of Messina, Messina, Italy

**Keywords:** photodynamic therapy, child, pediatric dermatology, acne vulgaris, viral warts, Gorlin syndrome, necrobiosis lipoidica, hidradenitis suppurativa

## Abstract

Photodynamic therapy (PDT) is a photochemotherapy based on local application of a photosensitive compound and subsequent exposure to a light source of adequate wavelength. It is a non-invasive therapeutic procedure widely used in oncodermatology for treatment of numerous skin cancers, but in the last years its use has been gradually extended to an increasing list of skin diseases of both infectious and inflammatory nature. Although PDT is proven as a safe and effective therapeutic option in adults, its use is not well standardized in the pediatric population. In this review, we will focus on clinical applications, mechanisms of action, protocols, and adverse events in children and adolescents. Most of pediatric experiences concerned treatment of skin cancers in Gorlin syndrome and xeroderma pigmentosum, acne vulgaris, and viral warts, but other applications emerged, such as cutaneous lymphoma and pseudo-lymphomas, necrobiosis lipoidica, hidradenitis suppurativa, dissecting cellulitis, leishmaniasis, angiofibromas, verrucous epidermal nevus, and linear porokeratosis. In these pediatric diseases, PDT appeared as an effective therapeutic alternative. The results on vitiligo were limited and not fully encouraging. Although highly versatile, PDT is not a therapy for all skin diseases, and a deeper knowledge of its mechanisms of action is required to better define its spectrum of action and safety in pediatric patients.

## Introduction

Photodynamic therapy (PDT) is an attractive, non-invasive therapeutic procedure widely used in adult patients for treatment of tumoral, inflammatory, and infectious skin diseases. PDT is a photochemotherapy based on local application of a photosensitive compound and subsequent exposure to a light source of adequate wavelength. The most employed photosensitizers commonly used in dermatology are the 5-aminolaevulinic acid (ALA, an intermediate of the heme biosynthetic pathway) and its methyl ester 5-aminolevulinate (MAL), which are converted inside the target cells to photo-active protoporphyrin IX (PpIX). After an incubation period (generally 3 h), PpIX is activated by an artificial light source (conventional PDT) or by sunlight (daylight PDT), thus leading to the production of reactive oxygen species (ROS), triggering both apoptosis and necrosis of target cells as well as stimulation of an immune modulating response. Different light sources with varying wavelengths can be used in PDT. The absorption spectrum of protoporphyrin IX shows maximal absorption peaks at approximately 410 nm, namely, at the wavelength of blue light, but it also shows smaller absorption peaks at 506, 532, 580, and 630 nm as well, namely, within the red light wavelength (Prieto et al., 2005). Nevertheless, the effect of red light appears to be stronger than that observed with blue light because of the greater depth of penetration of red light into dermis, thus explaining its diffuse use worldwide with respect to blue light. DL-PDT is a novel procedure in which the activation of the topical photosensitizer is induced by exposure to natural daylight, without requiring preliminary occlusion and dedicated instrumental equipment. With respect to the conventional one, DL-PDT has a more superficial depth of penetration, so its use is reserved to thin lesions (Borgia et al., 2020a).

The heterogeneous mechanisms of action and the multiple targets hit by PDT have allowed to progressively extend its use from the treatment of non-melanoma skin cancer to an always increasing list of skin diseases of both infectious and inflammatory nature. PDT displays several major strengths: it is a non-invasive, easily repeatable, outpatient treatment that can be applied to wide areas of affected skin with an overall good profile of safety. PDT can be used in fragile patients, that is, elderly subjects in whom surgery is contraindicated, in immuno-depressed subjects, or to treat large or multiple lesions localized in poor healing areas. Moreover, PDT shows superior cosmetic outcome compared with more invasive therapeutic approaches such as surgery and cryotherapy, with no scarring and pigmentary changes. Although PDT is proven as a safe and effective therapeutic option in several dermatologic diseases in adults, its use is not well standardized in pediatric population. For this reason, we performed a review about the employment of PDT in the pediatric age to provide an overview of the current state of art and to explore new potential fields of use of this technique.

## Methods

We checked the PubMed (https://ncbi.nlm.nih.gov/PubMed) database using the string “photodynamic therapy” [All Fields] AND “skin” [All Fields].

Only the research works written in English language, concerning humans and child (birth to 18 years), and with no time limits were included. A systematic literature search was led according to the PRISMA flowchart, also reviewing the abstracts of the articles whose title suggested this association. The references retrieved were critically examined by two experts in the field of dermatology to select those pertinent to our research, namely, clinical trials, retrospective studies, case series, and case reports. Reviews were excluded, but their reference lists were also examined to find other relevant articles, which were eventually revised and included if appropriate.

**FIGURE 1:**
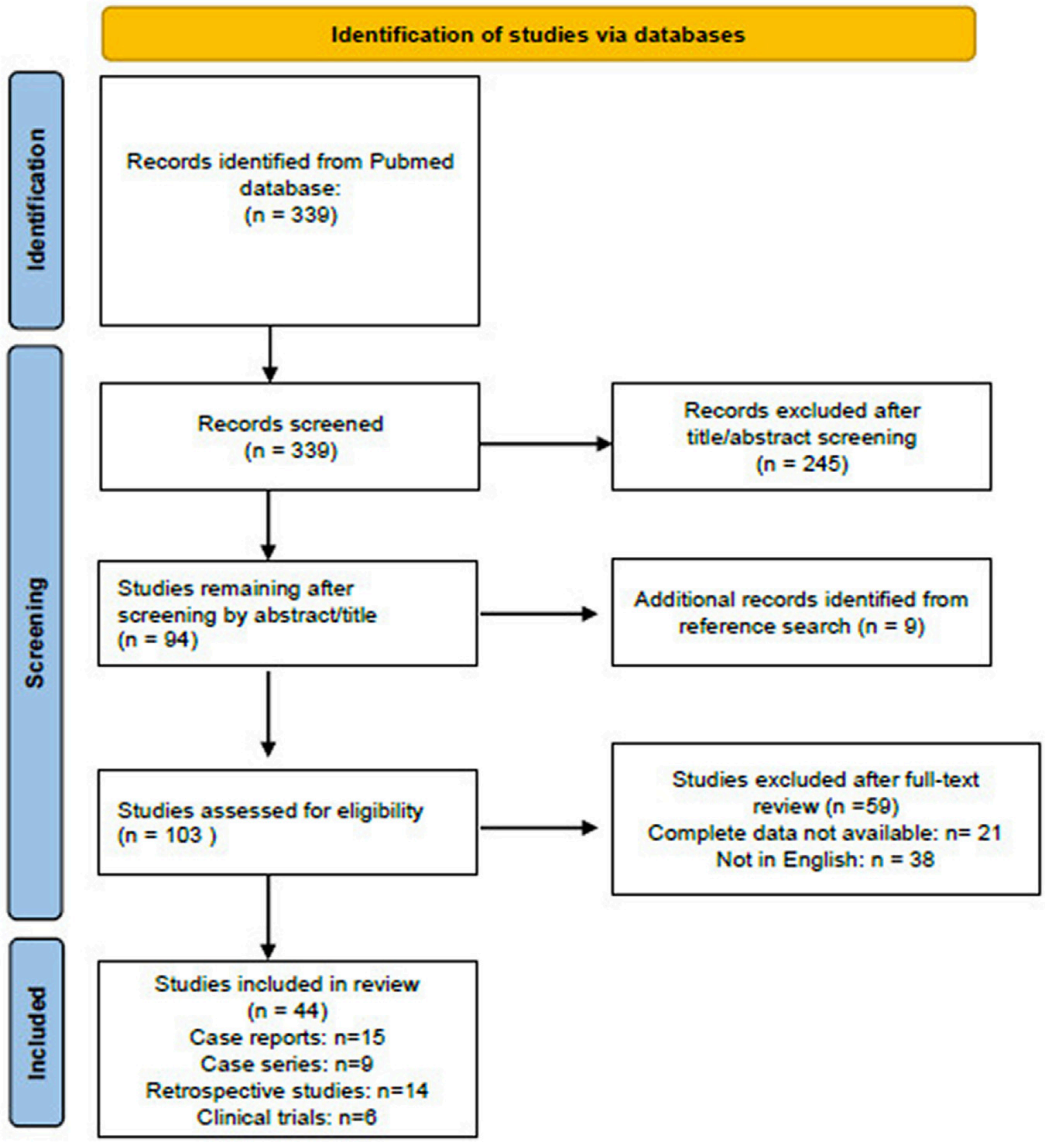
Flow diagram of the literature screened using the Preferred Reporting Items for Systematic Reviews and Meta-Analyses (PRISMA) guidelines. The figure is adapted from http://prisma-statement.org.

Figure 1 summarizes the publication screening scheme used according to PRISMA guidelines.

We included only studies on patients treated with topical ALA-PDT or MAL-PDT, the photosensitizers widely available and therefore most commonly used by dermatologists. As of 6 January 2022, 44 articles were identified. Studies exclusively focusing on pediatric patients were 33. Parameters of these studies, including patients’ features, type of topical photosensitizers and light sources used, conditions of treatment, number of treatments, and outcomes and adverse events, are summarized in Tables 1, 2. Studies including both adults and children with no specific data on pediatric patients were 11. They were also included in results and summarized in Tables 3, 4. For convenience, the results have been categorized into three main topics (oncologic, anti-inflammatory, and antimicrobial), with a fourth paragraph including a miscellanea mainly dealing with disorders of keratinization.

**TABLE 1:** Studies with specific data on pediatric patients. Patients’ age, clinical features, and PDT clinical outcomes.

DX	Publication (first author, year)	Site	Previous treatment	No ped pt	Age (y)	Clinical outcome	Adverse event
BCCs	Oseroff et al. (2005)	Anywhere	Surg, laser, topical and systemic retinoids, and ALA-PDT	3/3	6,10,17	85–98% CR	LSRs, pigm, and hair loss
AKs	Larson and Cunningham (2012)	Face	5-FU and imiq	1/1	16	Improvement	Not
BCCs and AKs	Fernández-Guarino et al. (2020)	Face	Imiq and surg	12/13	9.3, range 2–18	Improvement	LSRs
BD	Hyun et al. (2016)	Hand	—	1/1	12	CR	No
LBC	Takeda et al. (2005)	Face	Topical CCS	2/2	16	CR	Pigm
PgR	Mendese et al. (2012)	Foot	—	1/1	10	CR	Pain and pigm
MF	Heng et al. (2014)	Thigh	UVA1, topical PUVA, imiq, LEBT, CCS, and tazarotene	1/46	<16	CR	—
LyP	Snider et al. (2020)	Forearm	Topical and intralesional CCS, doxycycline, and nB-UVB	1/1	13	CR	—
Ang (TS)	Weinberger et al. (2009)	Face	—	2/6	10, 18	PR	LSRs
VEN	Zheng et al. (2018)	Face	Cryo	1/1	17	CR	—
LP	García-Navarro et al. (2009)	Lower leg	Topical calcipotriol	1/1	13	SR	LSRs
	Curkova et al. (2014)	Arm	Emollients	1/1	16	SR	LSRs
	Garrido-Colmenero et al. (2015)	Breast	Adapalene and topical tacrolimus 0.1%	1/1	11	PR	LSRs
Porokeratosis	Gracia-Cazaña et al. (2015)	Neck and popliteal fossa	Calcipotriol, 5-FU, and imiq	1/1	12	CR	—
Acne	Ma et al. (2015)	Face	—	21/21	16.17 ± 1.43	95.23% CR after 8 weeks	Pain, LSRs, and pigm
	Theresia et al. (2017)	Face	Topical and oral retinoids and antibiotics	1/4	17	Improvement	No
	Borgia et al. (2018a)	Buttocks	Oral minocycline and topical and oral retinoids	1/1	16	Improvement	Pain and LSRS
	Li et al. (2018)	Face	Oral retinoids and antibiotics	2/2	18	Improvement	Pain and LSRs
	Itoh et al. (2001)	Face	Yes	2/13	18	Improvement	LSRs and pigm
	Hörfelt et al. (2007)	Face and back	Tetracyclines	4/15	16.5	Improvement	Pain and pigm
NL	Berking et al. (2009)	Lower legs	—	3/18	16.6, range 16–17	1/3 PR and 2/3 NR	Pain
HS	Bu et al. (2017)	Armpit and head and neck	—	2/7	15, 16	Improvement DLQI	Pain
DC	Feng et al. (2019)	Scalp	Oral and topical antibiotics	1/8	15	Improvement	Pain and LSRs
PCAS	Cui et al. (2020)	Scalp	Incision, drainage, and systemic antibiotics	1/9	17	SR	—
Vg	Zhang et al. (2018)	Abdomen and forehead	—	2/2	17, 4	NR (pt A), improvement (pt B)	—
CVW	Borgia et al. (2020a)	Foot	Cryo	1/1	6	CR	LSRs
	Huang et al. (2019)	Plantar regions	Cryo, laser, and 5-FU	1/1	18	CR	Pain
	Wu et al. (2019)	Hand	Cryo, laser, imiq, and intralesional 5-FU	10/23	14.5, range 5–18	90% CR	Pain, LSRs, and onychodystrophy
FVW	Borgia et al. (2019)	Face	Topical retinoid and cryo	1/1	8	CR	Pain and pigm
	Borgia et al. (2020a)	Face	Conventional therapies	30/30	9.67 (gp A), 9.13 (gp B)	73.3% CR (C-PDT),80% CR (DL-PDT)	
GVW	Xu et al. (2018)	Perianal and intra-anal	Imiq and cryo	8/8	1.8, range 1–4	CR	Pain
	Macca et al. (2022)	Perineal		1/1	5	CR	LSRs and pigm
CL	Johansen et al. (2019)	Lower leg	MA and topical and oral antimycotics	1/1	15	CR	Pain

**TABLE 2:** PDT protocols.

DX	Publication (name first author and year)	PS	PS incub (h)	Light source	Light dose (J/cm^2^)	Mean no. of treat	Associat therapy	Follow-up (w, m, y)
BCCs	Oseroff et al. (2005)	20% ALA	18–24	633 ± 2 nm (laser) and 590–700 nm (lamp)	60–240	1–3	—	6 years
AKs	Larson and Cunningham (2012)	ALA	40 min	417–432 nm (lamp)	—	3	—	1 year
BCCs and AKs	Fernández-Guarino et al. (2020)	ALA and MAL	—	Light indoor	—	1	—	3 m
BD	Hyun et al. (2016)	MAL	3	Red light	—	2	—	9 m
LBC	Takeda et al. (2005)	20% ALA	4–6	630 and 700 nm visible light (lamp)	120	5	—	6 m
PgR	Mendese et al. (2012)	20% ALA	2	417 ± 5 nm (lamp) and 595 nm (PDL)	10–11.5	9	—	46 m
MF	Heng et al. (2014)	MAL	—	—	—	30	—	35 m
LyP	Snider et al. (2020)	20% ALA	1	Blue light (lamp)	10	3	—	2 m
Ang (TS)	Weinberger et al. (2009)	20% ALA	1	417 nm (lamp)	10	3–4	PDL	
VEN	Zheng et al. (2018)	20% ALA	4	635 nm (semiconductor laser)	120	4	Fmp-RF	24 w
LP	García-Navarro et al. (2009)	16% MAL	3	630 nm (LED)	37	2	Shaving	11 m
	Curkova et al. (2014)	16% MAL	3	630 nm (LED)	37	3	Curett	1 year
	Garrido-Colmenero et al. (2015)	16% MAL	2	630 nm (LED)	37	2	—	4 m
Porokeratosis	Gracia-Cazaña et al. (2015)	MAL	3	630 nm (LED)	37	3	—	5 years
Acne	Ma et al. (2015)	5% ALA	1	633 ± 10 nm (LED)	90–96	3	—	8 w
	Theresia et al. (2017)	5–10% ALA	2–3	633 ± 10 nm (LED)	96–180	4	—	6 m
	Borgia et al. (2018b)	10% ALA	3	630 nm (diode)	75	6	—	6 m
	Li et al. (2018)	3% ALA	3	633 ± 6 nm (LED)	50	3	—	2 years
	Itoh et al. (2001)	20% ALA	4	600 ± 700 nm (lamp)	13	1	—	6 m
	Hörfelt et al. (2007)	20% ALA	3	635 nm (lamp)	30–70	1	—	10 w
NL	Berking et al. (2009)	16% MAL	3	630 nm (LED)	37	2–6	—	4–8 w
HS	Bu et al. (2017)	20% ALA	3	635 nm (laser)	—	4	—	6–12 m
DC	Feng et al. (2019)	10% ALA	3	633 ± 10 nm (LED)	96–180	3	p-bn	
PCAS	Cui et al. (2020)	20% ALA	2	635 nm (laser)	80	1	Reverse flap	6 m
Vg	Zhang et al. (2018)	1.5% ALA	3	633 ± 10 nm (semiconductor laser)	96	23	—	2 years
CVW	Borgia et al. (2020b)	10% ALA	3	630 nm (LED)	75	2	—	1 year
	Huang et al. (2019)	10% ALA	4	635 nm (semiconductor laser)	—	3	Shaving	16 m
	Wu et al. (2019)	10% ALA	3	633 nm red light source	—	3	Shaving	12 m
FVW	Borgia et al. (2019)	10% ALA	0.5	Sunlight	—	2	No	1 year
	Borgia et al. (2020b)	10% ALA	3 h (C-PDT), 0.5 h (DL-PDT)	630 nm (diode) for C-PDT and sunlight for DL-PDT	75 (C-PDT group)	3	No	24 w
GVW	Xu et al. (2018)	20% ALA	4	635 nm (semiconductor laser)	—	6	Microwave ablation	6 m
	Macca et al. (2022)	10% ALA	3	630 nm (LED)	75	3		6 m
CL	Johansen et al. (2019)	10% ALA	—	—	—	24		1 m

**TABLE 3:** Studies including both adults and children with no specific data on pediatric patients. Patients’ age, clinical features, and PDT clinical outcomes.

DX	Publication (name first author and year)	Site	Previous treatment	No pt	Age (y)	Clinical outcome	Adverse event
BCCs	Loncaster et al. (2009)	All body	surgery, cryo, and 5-FU	33	40, range 9–79	Control rates of 73.0% for lesions <1 mm and 40.8% for lesions between 1 and 2 mm	—
Acne	Hörfelt et al. (2006)	Face	—	30	18, range 15–28	54% mean reduction (inflamed lesions)	Pain and LSRs
	Wang et al. (2010)	Face	Topical and systemic antibiotics, oral retinoids, and sytemic steroids	78	22.9, range 16–37	90% clearance in 90% of patients	Pain, LSRs, and pigm
	Barolet and Boucher (2010)	Face and back	—	10	26.2, range 13–54	73% median reduction (inflamed lesions)	Pain, LSRs, and acneiform folliculitis
	Xu et al. (2017)	Face	—	95	24, range 15–35	74.4% mean reduction of inflamed lesions and 61.7% of non-inflamed lesions	Pain and LSRs
	Rojanamatin et al. (2006)	Face	—	14	16–27	8.7% decrease in lesion counts	Pain, LSRs, and pigm
	Orringer et al. (2010)	Face	—	44	25, range 15–50	30% (inflamed lesions) and 7% (non-inflamed lesions) improvement	LSRs and pigm
NL	Kaae et al. (2018)	Lower legs	Topical corticosteroids and cryotherapy	65	35.5, range 12–65	80% CR (DL-PDT) and 64% CR (MAL-PDT)	Pain
CVW	Schroeter et al. (2005)	Plantar region	Surg, cryo, sa, silver nitrate, and 5-FU	31	29, range 6–74	88% CR	Pain, LSRs, and pigm
	Caccavale et al. (2019)	Hand and plantar region	cryo, etc	13	28.8, range 18–52	84.6% CR	Pain, LSRs, and pigm
GVW	Shan et al. (2016)	Male urethra	—	76	32.6, range 16–65	93.4% CR	Pain and LSRs

**TABLE 4:** PDT protocols.

DX	Publication (name first author and year)	PS	PS incub (h)	Light source	Light dose (J/cm2)	Mean no. of treat	Associat therapy	Follow-up (w, m, y)
BCCs	Loncaster et al. (2009)	16% MAL or 20% ALA	6	630 ± 15 nm (LED)	100	1–3	—	12–24 m
Acne	Hörfelt et al. (2006)	16% MAL	3	635 nm (lamp)	37	2	—	3 m
	Wang et al. (2010)	10% ALA	3	633 nm (LED)	50–70	1–3	—	6 m
	Barolet and Boucher (2010)	20% ALA	1	630 nm (LED)	70	1	Radiant infrared	1 m
	Xu et al. (2017)	5% ALA	1.5	633 nm (LED)	120	4	Oral minocycline	8 w
	Rojanamatin et al. (2006)	20% ALA	0.5	560–590 nm (IPL)	30	3	—	12 w
	Orringer et al. (2010)	20% ALA	1–1.5	PDL	6.5–7.5	1–3	—	16 w
NL	Kaae et al. (2018)	16% MAL	0.5–3	634 nm (diode), sunlight	37	4	Curettage	14 m (range 2–81)
CVW	Schroeter et al. (2005)	20% ALA	6.8	400–450 nm,580–720 nm (halogen)	—	2.3	Blunt scraping	3 m
	Caccavale et al. (2019)	10% ALA	3	630 nm red light source	75	3	Curettage, microneedling	Mean 4.3 m
GVW	Shan et al. (2016)	20% ALA	4	635 nm (laser)	—	1–4	—	3 m

Abbreviations: 5-FU, 5% topical 5-fluorouracil cream; Age (y), age of pediatric patients in years (Table 1); Age (y)= age of patients in years (Table 3); Ang TS, angiofibromas of tuberous sclerosis; Associat therapy, associated therapy; BD, Bowen's disease; CCS, corticosteroid; CL, cutaneous leishmaniasis; CR, complete response; cryo, cryotherapy; Curett, curettage; CVWs, cutaneous viral warts; DC, dissecting cellulitis; DX, diagnosis; Fmp-RF, fractional micro-plasma radiofrequency; Follow-up (w, m, y), Follow-up (weeks, months, years); FVWs, flat viral warts; gp, group; GVWs, genital viral warts; HS, hidradenitis suppurativa; imiq, imiquimod cream; LBC, lymphadenosis benigna cutis; LEBT, localized electron beam therapy; LP, linear porokeratosis; LSRs, local skin reactions, including erythema, burning, edema, crusting, desquamation, or pustule; LyP, lymphomatoid papulosis; MA, meglunime antimoniate; Mean no. of treat., mean number of treatments; min: minutes; NL, necrobiosis lipoidica; No pt, no. patients submitted to PDT (Table 3); No. ped pt, No. of pediatric/total patients submitted to PDT (Table 1); NR, no response; p-bn, plum blossom needle; PCAS, perifolliculitis capitis abscedens et suffodiens; PDL, pulsed dye laser; pigm, pigmentation changes, including hypo/hyperpigmentation; PgR, pagetoid reticulosis; PR, partial response; PS incub (h), photosensitizer incubation (hours); PS, photosensitizer; pt, patient; PT, physical therapy; sa, salicylic acid; SI, significant improvement; SR, satisfactory response; surg, surgery; VEN, verrucous epidermal nevus; Vg, vitiligo.

## PDT and pediatric skin cancers

The onset of skin cancers in pediatric age is a rare event. Nevertheless, some genetic syndromes, such as Gorlin syndrome or xeroderma pigmentosum, may predispose to the development of skin tumors since childhood.

### Gorlin syndrome

Gorlin syndrome, or nevoid basal cell carcinoma syndrome (NBCCS), is an autosomal dominant syndrome caused by mutations in the PTCH1 (Patched 1) gene, characterized by multiple basal cell carcinomas (BCCs) occurring from puberty, in addition to various dental, osseous, ophthalmic, neurological, and sex organ abnormalities (Jawa et al., 2009).

A total of three Gorlin patients <18 years old (6, 10, and 17 years) with diffused BCCs were treated by means of ALA-PDT, achieving 85 to 98% complete response (Oseroff et al., 2005).

Oseroff et al. (2005) performed several sessions of 10% ALA-PDT on the children with a red light laser for areas with lower diameter (from 2 to 7 cm) and a lamp for those with larger diameter (up to a 16 cm diameter). The patients presented BCCs on 12–25% of their body surface, and four to seven sessions were needed for each patient but individual areas received one to three treatments. The patients reported excellent cosmetic outcomes with no evidence of new BCCs in the treated areas up to 6 years of follow-up.

Loncaster et al. (2009) treated 33 Gorlin patients of all ages (range 9–79 years) with ALA-PDT and MAL-PDT, obtaining different control rates depending on the thickness of lesions. They performed ultrasound investigation to assess lesion thickness and used topical PDT to treat only superficial lesions (<2 mm thick). Thicker lesions were treated with a systemic photosensitizer. At 12 months, local control rates were 73.0% for lesions <1 mm, 40.8% for lesions measuring between 1 and 2 mm, and 59.3% for lesions >2 mm.

Gorlin patients are highly susceptible to DNA damage from therapies such as ionizing radiation. However, Oseroff et al. (2005) found no evidence of ALA-PDT inducing or promoting BCCs in pediatric patients. Gorlin patients have increased risk of medulloblastoma and may develop multiple to thousands BCCs in the site of radiotherapy, namely, on the anterior surface of abdomen and on the back. In these cases, the carcinomas are so numerous that surgical excision is impractical. There are reports of pediatric Gorlin patients who benefited from PDT for the treatment of radiotherapy-induced BCCs (Walter et al., 1997; Oseroff et al., 2005). In adult patients, MAL-PDT or nanoemulsion ALA-PDT are considered for non-aggressive, low-risk BCC, that is, small superficial and nodular types not exceeding 2 mm tumor thickness, where surgery is impractical or contraindicated, or avoidance of scarring is a priority (Rhodes et al., 2004; Peris et al., 2019). Ultrasound may be useful to assess BCC thickness prior to treatment and assign Gorlin patients to a PDT treatment, as demonstrated by Loncaster et al. (2009). An international experts consensus established recommendations for the use of MAL-PDT in patients with Gorlin syndrome (Basset-Seguin et al., 2014). Although MAL-PDT is not approved for children, three of the seven expert panel members had pediatric experience and all agreed that MAL-PDT might also be considered in the pediatric age (Basset-Seguin et al., 2014).

A recent study in adult patients observed higher recurrence rates in BCCs of neck and head treated with MAL-PDT (Condorelli et al., 2022). Vinciullo et al. highlighted that lesions located in the H-zone, whether large or not, had unfavorable CCR following MAL-PDT (Vinciullo et al., 2005). For these reasons, according to the most recent European guidelines for treatment of BCC, less common histologic variants of BCC, such as morphoeic, pigmented, and micronodular types, as well as areas with higher risk of tumor survival and deep penetration (facial ‘H’-zone) should not be treated with PDT (Peris et al., 2019). Usually, PDT for pigmented BCC treatment is not performed because of the lower penetration of the light, possibly due to the melanin content of these variants (Salvio et al., 2021). During the treatment of their pediatric patients with Gorlin syndrome, Oseroff et al. (2005) noted that a patient presented a lower response rate probably due to the varying pigmentation of his BCCs, which reduced the effective light dose. A prior debulking of pigmented BCC with removal of pigmented component may be an option for the treatment of these subtypes. Its suitability should also be investigated in pediatric population. In a group of adult patients, Salvio et al. (2021) performed debulking of 30 pigmented BCC before MAL-PDT and obtained complete response in 100% of cases, with no recurrence at mean 24-month follow-up.

### Xeroderma pigmentosum and Bowen’s disease

Xeroderma pigmentosum (XP) is a rare autosomal recessive disorder of defective UV-radiation–induced damage repair that is characterized by photosensitivity and higher risk for developing skin cancer at an early age (Black, 2016). Reports of PDT in XP are scarce because of the fear of developing skin cancers following illumination (Fernández-Guarino et al., 2020). Larson and Cunningham successfully treated facial actinic keratosis of a 16-year-old girl with type C XP, by means of three sessions of ALA-PDT with blue light. Before starting treatment, Larson and Cunningham tested skin photosensitivity of their patient performing a 3 cm^2^ test treatment on her left arm, not noticing adverse cutaneous reactions of the exposed area. The treatment was repeated for 1 year and did not cause any adverse events (Larson and Cunningham, 2012). Fernández-Guarino et al. successfully treated 13 young African XP patients, 12 of whom were younger than 18, affected by facial AKs and BCCs, by using one session of DL-PDT. They used indoor DL-PDT because the window blocked UVB radiation, responsible for DNA damage, thus limiting treatment-related skin cancer induction. After two days of treatment, the patients presented with crusting and scaling, but a week later the cutaneous reaction resolved. No adverse events were noted at 3-month follow-up (Fernández-Guarino et al., 2020). Despite these encouraging results, the use of PDT in pediatric patients with XP is still limited and its safety should be confirmed by further studies considering long-term follow-up. Keratinocyte-derived tumors are rare in the pediatric age with a single case of periungueal Bowen’s disease treated with PDT in a 12-year-old boy. Hyun et al. treated the child with two sessions of MAL-PDT at an interval of 3 weeks. The authors did not observe any sign of recurrence 9 months after treatment, and no adverse events were reported (Hyun et al., 2016).

### Cutaneous lymphomas

Cutaneous lymphomas have a heterogeneous clinical presentation, ranging from pink, red, or violaceous solitary papules or nodules to widespread infiltrative lesions. There is an emerging interest about the antitumor properties of PDT applied to cutaneous lymphomas, particularly T-cell lymphomas, although only a few cases in adults, and even less in children, are described in literature.

Overall, five pediatric patients with various cutaneous lymphomas have been treated with MAL- or ALA-PDT, achieving a complete response in all cases (Takeda et al., 2005; Mendese et al., 2012; Heng et al., 2014; Snider et al., 2020).

Takeda et al. observed complete resolution of periocular lymphadenosis benigna cutis (also called lymphocytoma cutis) in two 16-year-old girls after five sessions of ALA-PDT. They noted only a transient hyperpigmentation and no recurrences at 6-months follow-up (Takeda et al., 2005). Mendese et al. treated a 10-year-old boy with pagetoid reticulosis, a rare variant of mycosis fungoides (MF), on his right foot by nine sessions of PDT over 13 months. They applied ALA topical solution with subsequent illumination with blue light or pulsed day laser. On three occasions, ALA was injected intralesionally to ensure adequate penetration. The patients reported treatment-related moderate pain and post-inflammatory pigmentation. At the end of the treatment period, he was clinically disease-free. At a follow-up visit 15 months after the last PDT session, two punch biopsies confirmed the absence of atypical lymphocytes in the treated area. Clearance was maintained at 46 months follow-up (Mendese et al., 2012). Heng et al. also reported a case of successful treatment of solitary mycosis fungoides after 30 sessions of MAL-PDT. After two months of stopping PDT, the patient showed no evidence of MF. The patient was disease-free at 35-months follow-up (Heng et al., 2014). Snider et al. used PDT in combination with narrow-band UVB to treat a 13-year-old boy with multiple lesions of lymphomatoid papulosis (LyP) over his elbows, forearms, proximal thighs, and right hip. They used 20% ALA topical solution and LED illumination to treat right forearm nodules resistant to nb-UVB treatment. He achieved the clearance of all lesions on his right arm within 2 months of combination therapy. Nevertheless, 2 years later new lesions appeared, but further PDT sessions were not attempted and subsequently the patient was lost at follow-up (Snider et al.,. 2020).

## PDT and pediatric inflammatory skin diseases

### Acne vulgaris

Acne is a chronic inflammatory disease of the sebaceous-pilosebaceous unit. In the last years, it has been clearly demonstrated that acne development is linked to the combination of predisposing genetic factors and environmental triggers, among which a prominent role is played by the follicular colonization by *Propionibacterium acnes* (*P. acnes*) (Antiga et al., 2015). Studies focusing exclusively on acne and PDT in pediatric population are scarce. In total, three possible ways of action have been proposed to explain the improvement of acne by PDT: photodynamic killing of *Propionibacterium acnes*, which sterilizes the sebaceous follicle; direct photodynamic injury of sebaceous glands inhibiting sebum production; and reduction of follicular obstruction by an effect on keratinocyte shedding and hyperkeratosis. Nevertheless, these mechanisms do not appear to occur simultaneously in all cases of acne improvement. In a study on 10 patients involving a 16-year-old female, Pollock et al. (2004) found a statistically significant reduction of inflammatory acne lesions following ALA-PDT but failed to demonstrate changes in *P. acnes* numbers or in sebum excretion in the same patients. It has been hypothesized that light destroys *P. acnes* by targeting its endogenous porphyrins, including coproporphyrin III and protoporphyrin (Yeung et al., 2007). Nevertheless, according to some studies, PDT may determine a functional damage of *P. acnes* rather than a quantitative reduction (Pollock et al., 2004; Hörfelt et al., 2007). Moreover, irreversible damage of sebaceous glands may be reached only with repeated session of PDT (Pollock et al., 2004). So, other PDT effects, such as the reduction of follicular obstruction or anti-inflammatory effects, may play a more important role for acne improvement than destruction of sebaceous glands or killing of *P. acnes* (Pollock et al., 2004; Hörfelt et al., 2007).

Overall 31 pediatric patients with acne vulgaris were treated with various concentrations of ALA.

Ma et al. (2015) carried out a prospective study to evaluate ALA-PDT in severe acne of 21 adolescent patients. They treated patients with an average of three PDT sessions. They obtained high rates of effective response (85.7%) and observed that the efficacy of ALA-PDT tends to increase even after, reaching 95.23% of effective response after 8 weeks. PDT appears particularly useful in acne treatment when other conventional therapies have failed. Theresia et al. (2017) treated a 17-year-old male patient with severe multiple nodulocystic acne lesions on the face with ALA-PDT, who failed treatment with numerous topical and oral retinoids and antibiotics. The authors noticed a mild reduction in inflammatory acne lesions and sebum secretion already after the first session of PDT. After the fourth session of ALA-PDT, the patient had long-term remission with no new lesions during the 6 months follow-up. Borgia et al. (2018a) obtained improvement and a sustained good response in a case of acne conglobata on the buttocks in a 16-year-old boy, who did not respond to oral minocycline, topical retinoid, and systemic isotretinoin. They used ALA-PDT for a total of six sessions. The patient experienced intense pain and inflammation during the first two sessions, then discomfort was milder. At the end of the treatment period, healing of the cutaneous nodules was observed, and at 6 months follow-up, the patient maintained good cosmetic results with no side effects. In total, two 18-year-old identical male twins with severe nodulocystic facial acne resistant to oral retinoids, antibiotics, and previous physical therapies experienced decrease in acne lesions with sustained response at 2-year follow-up after three sessions of ALA-PDT at 2 weeks interval. Both the patients suffered transient moderate pain and mild erythema during treatment with no residual pigmentation. They evaluated this method better than the previous medications (Li et al., 2018). In literature, several clinical trials or case series on PDT efficacy for acne treatment included both children and adults. Overall, 271 adult and pediatric patients with acne vulgaris were treated with various concentrations of ALA, achieving improvement of various degree, ranging from resolution of 30% of inflamed lesions and 7% of non-inflamed lesions (Orringer et al., 2010) to 90% clearance in 90% of patients (Wang et al., 2010). Itoh et al. tested the effects of 20% ALA oil-in-water emulsion and subsequent illumination by polychromatic visible light source on three men and 10 women with intractable facial acne. Among these, two 18-year-old males were also treated with one session of ALA-PDT and obtained excellent control of their acne and transient side effects. One of them experienced subsequent hyperpigmentation and was therefore treated with 4% hydroquinone cream for 10 days (Itoh et al., 2001). In a study on the efficacy of 20% ALA-PDT involving 15 patients with facial and dorsal acne vulgaris, four of them were pediatric (two of 16 years and two of 17 years). Improvement of acne lesions were recorded in all pediatric patients with facial acne after one session of ALA-PDT. There were no data about the 17-year-old male affected by dorsal acne because he was lost at 20-weeks follow-up (Hörfelt et al., 2007).

In a RCT on both pediatric and adult patients (median 18 years, range 15–28), two sessions of MAL-PDT, 2 weeks apart, determined a median reduction of 54% in the total inflammatory lesion count at week 12. Clinical response in MAL-PDT–treated patients was significantly better with respect to placebo-treated patients. Nevertheless, MAL-PDT was associated with more pain than placebo-PDT (Hörfelt et al., 2006). Wang et al. evidenced a 90% clearance rate after three sessions of ALA-PDT in a group of patients with mean age 22.9 years (range 16–37). Only 10% of patients had clearance rates between 50 and 90%. Side effects were well tolerated and transient, except for a patient who left the study because of excessive pain and discomfort (Wang et al., 2010). In a study involving patients with an age range 13–54 years, Barolet and Boucher et al. increased skin temperature of 10 patients with radiant infrared (IR) prior to ALA-PDT application to enhance the PS penetration. The authors observed a significant difference in median reduction of inflammatory lesions on the IR pre-treated vs. the control side 1 month after PDT. The authors did not report unusual treatment–related adverse effects (Barolet and Boucher, 2010). Also the combination of PDT and antibacterial therapies for acne has been investigated with encouraging early results. In a clinical trial, Xu et al. compared the effects of minocycline plus ALA-PDT and minocycline alone on moderate-to-severe facial acne of 95 patients aged 15–35 years. The authors observed a greater mean percentage reduction of lesion counts in the minocycline plus PDT group compared to the minocycline-alone group at 8 weeks follow-up for both inflammatory and non-inflammatory lesions and only mild and transient adverse events in the minocycline plus PDT group (Xu et al., 2017). Intense pulsed light (IPL) has been investigated as a light source in PDT for juvenile acne. Rojanamatin et al. treated 14 patients (range 16–27 years) with topical ALA plus IPL for three sessions. The combination determined a decrease in lesions counts of 87.7% at 12 weeks (Rojanamatin et al., 2006). Also, a pulsed dye laser (PDL) has been evaluated as a light source for ALA-PDT. Orringer et al. treated 44 patients, including pediatric patients (mean age 25 years, range 15–50) with three PDL treatments after a 60–90 min ALA application time. Nevertheless, the results with this light source were not particularly exciting with only transient decrease in mean inflammatory papule counts but no statistically significant differences in lesion counts (papules, pustules, and open and closed comedones) between treated and untreated control skin at the conclusion of the study on week 16 (Orringer et al., 2010). However, compared to topical or systemic treatments for juvenile acne, PDT did not show clear advantages in efficacy, and for this reason, it should be reserved for severe, recalcitrant cases, resistant to antibiotics and/or hormonal conventional treatments and/or not eligible to treatment with isotretinoin. In a systematic review on light therapies for acne, 25 trials on a total of 694 patients, also including pediatric patients, were analyzed. The authors observed that PDT did not show better efficacy than topical 1% adapalene gel. Nevertheless, PDT showed a benefit over light therapy alone (Hamilton et al., 2009).

### Necrobiosis lipoidica

Necrobiosis lipoidica (NL) is a rare granulomatous disease strongly, but not exclusively, associated with diabetes mellitus characterized by yellowish-brown telangiectatic plaques with central atrophic area and erythematous edge, usually localized on the pretibial skin of females. Treatment of NL is often not satisfactory. PDT exerts positive effects in controlling the disease. In addition to its general anti-inflammatory effects, PDT seems to influence positively the course of NL by remodeling the collagen matrix, thus stimulating the wound healing and improving sclerosis (Borgia et al., 2014). PDT is able to induce matrix metalloproteinases (MMP) production in fibroblasts (Motta and Monti, 2007) with higher production of MMP-1, MMP-9, and transforming growth factor (TGF)-β3 in wounds treated with MAL-PDT compared to untreated wounds (Mills et al., 2014). Berking et al. obtained partial results from the treatment of three adolescents among 18 patients with necrobiosis lipoidica of lower legs. The patients received two to six sessions of MAL-PDT. Pain was measured by using a 10 cm visual analog scale and pediatric patients referred a pain level ranging from 4/10 to 10/10. One of them stopped the treatment after two sessions because of treatment-related pain achieving no benefit. Of the remaining two patients, one presented partial response, whereas the other was not responder (Berking et al., 2009). Kaae et al. (2018) conducted a retrospective study on 65 NL patients treated with DL-PDT and C-PDT, including pediatric patients (median age at first treatment 35.5 years, range 12–65) and observed complete response in 66% of cases, with similar rates between C-PDT and DL-PDT. MAL-PDT was in median performed four times. The authors observed no correlation between clinical response and gender, age at first PDT treatment, duration of NL prior to PDT treatment, number of NL elements, or diabetes.

### Hidradenitis suppurativa and dissecting cellulitis (perifolliculitis capitis abscedens et suffodiens or PCAS)

HS, often designed as acne inversa, is a chronic follicular inflammatory skin disease characterized by characterized by occlusion of hairs follicles and inflammation, which clinically leads to painful nodules, abscesses, and interconnecting sinus tracts involving the axillary, inguinal, anogenital, and inframammary regions. It usually manifests after puberty, but it can affect young patients especially those with familial history of HS. Bu et al. used 20% ALA-PDT as adjuvant therapy post-surgery for HS in seven patients, including two adolescents of 15 and 16 years, with Hurley grade II and III, respectively. At 5 months, they observed a marked improvement of the Dermatology Life Quality Index (DLQI) in all patients, including pediatric patients, with no recurrences at 6–12 months follow-up. Pain during illumination was well tolerated in all cases, but the 16-year-old patient, with Hurley Grade III lesions on craniofacial and neck areas, took the analgesic 30 min before PDT due to the moderate pain (Bu et al., 2017).

PCAS is considered an inflammatory bacterial process frequently caused by *Staphylococcus aureus* and *Staphylococcus epidermidis*. Cui et al. speculated that PDT ameliorated PCAS by inhibiting bacterial infection. In their case series of nine patients treated with combination of surgical reverse flaps and PDT, they included a 17-year-old-boy, who reported satisfied outcome after a single session (Cui et al., 2020). Feng et al. (2019) treated a 15-year-old boy among eight male patients with dissecting cellulitis by using ALA-PDT. Before 10% ALA application and red light illumination, they cut hair and performed micropunctures in skin lesions by using a plum blossom needle, a kind of micro-needle. After 3 months, the authors observed a significant improvement in the pediatric patient, with a clearance rate > 70% and marked relief of symptoms. Treatment-related pain was tolerable and previous analgesia was not needed.

## PDT and pediatric infectious skin diseases

PDT exerts antimicrobial effects on viruses, bacteria, fungi, and parasites. First, such activity is related to its ability to form high amount of reactive oxygen species (ROS), which damage biomolecules of all type of microorganisms, including viruses (Pérez-Laguna et al., 2018). In addition, as for tumoral cells, PDT stimulates the recognition of microorganisms by the immune system and mediates a local immune response against them. HPV is one of the most frequently targeted viruses in pediatric PDT. Cells infected by HPV are ideal targets of PDT because high-proliferating and can selectively accumulate PpIX compared to the surrounding non-infected cells. Moreover, selective photosensitization occurs not only in clinical HPV lesions but also in subclinical infection (Shan et al., 2016). Viruses have no capacity for PPIX production, but it has been demonstrated that the addition of exogenous ALA and its derivatives induces selective accumulation of PPIX in HPV-infected cells. PDT is able to significantly reduce HPV viral loads and to promote viral inactivation *via* cell necrosis and induction of T lymphocyte–mediated immune response against infected keratinocytes by increasing levels of IFN-α and IFN-ß (Borgia et al., 2020a). In bacterial infection, PDT-induced oxidative stress may damage multiple targets such as DNA, membrane integrity, protease activity, and lipopolysaccharide (LPS). Nevertheless, Gram-positive bacteria appear more sensitive to PDT than Gram-negative. Peptidoglycans and lipid acids in wall of Gram-positive allow penetration of cationic, anionic, and even neutral PS, while the double membrane in Gram-negative is particularly hampering and only cationic PS are active against them. Nevertheless, new technologies, including nanoparticle-based PDT, have significantly increased the PS penetration. The onset of resistance to PDT in bacteria is very unlikely because PDT-induced oxidative stress does not have a specific target but causes destruction of cell in different ways. Therefore, PDT has several advantages to antibiotics and may be considered a valid therapeutic alternative to them (Pérez-Laguna et al., 2018).

### Cutaneous viral warts

PDT is indicated for treatment of cutaneous viral warts when other therapies have failed or in difficult to treat cases.

Overall 12 pediatric patients with cutaneous viral warts were treated with various concentrations of ALA, achieving complete response in almost all cases (Borgia et al., 2020b; Huang et al., 2019; Wu et al., 2019).

Borgia et al. described the case of a 6-year-old girl with multiple viral warts on the dorsal left foot. After failure of cryotherapy, the authors performed ALA-PDT for two sessions, 1 month apart. In each session, the patient experienced mild burning sensation. Complete clearance of the treated warts was seen 6 weeks after the second treatment with no recurrences at 1-year follow-up (Borgia et al., 2020b). Huang et al. described the exciting case of an 18-year-old female who completely resolved her 2 year history of resistant multiple warts in the right foot after curettage plus PDT. The patient was treated with ALA-PDT for a total of three sessions, but superficial shaving was applied only for the first session. At 3-months follow-up, the warts disappeared with no residual scar (Huang et al., 2019). Wu et al. performed on average three sessions of ALA-PDT on 10 pediatric patients ranging from 5 to 18 years among 23 total patients with multi-resistant periungual warts. The patients underwent superficial shaving before the first PDT, not performed in the following additional sessions of PDT. The authors observed an overall complete clearance in 61% of patients with a higher rate in the pediatric group (9/10 young patients had complete response, namely, 90%). All patients completed the treatment and satisfactory cosmetic outcome was obtained in almost all patients (96%). A significant decrease in DLQI at 12-month follow-up was reported. Pain was the most common adverse event, followed by secondary onychodystrophy, mild itching, and blisters (Wu et al., 2019). Also, in case of cutaneous viral warts, many studies on PDT efficacy include both children and adults.

Overall, 44 adult and pediatric patients with cutaneous viral warts were treated with 10–20% ALA, achieving complete response in 84.6–88% of cases (Schroeter et al., 2005; Caccavale et al., 2019).

Schroeter et al. (2005) treated 48 plantar warts from 31 patients (mean age 29 years, range 6–74) with 20% ALA cream and red light, observing a complete response in 88% of cases and no significant side effects. Caccavale et al. (2019) proven the efficacy of combination of curettage plus microneedling plus topical ALA-PDT for the treatment of acral resistant warts in young patients (mean age 28.8 years, range 18–52). They performed a thorough curettage on palmar and plantar warts of 13 patients, subsequent application of 10% ALA cream and microneedling. After 3 h of incubation, the warts were irradiated with a red light source. After three sessions of treatment, at 3-week intervals, the authors observed complete remission in 84.6% of cases and partial remission in further 7.7%.

### Flat viral warts

PDT should be considered a useful option in treatment of flat warts, particularly in aesthetically sensitive areas such as the face of children (Borgia et al., 2020a). Overall 31 pediatric patients with flat viral warts were treated with C-PDT or DL-PDT, achieving complete response in one case (Borgia et al., 2019) and various degree of complete response ranging from 73.3% (C-PDT) to 80% (DL-PDT) in a study involving 30 patients (Borgia et al., 2020a).

Flat viral warts appear to be responsive not only to conventional PDT but also to DL-PDT. In the case of an 8-year-old female child with multiple facial flat warts resistant to previous topical tretinoin and cryosurgery, Borgia et al. performed two sessions of DL-PDT with 10% ALA ointment obtaining complete response with no recurrence at 1 year follow-up (Borgia et al., 2019). Borgia et al. also compared efficacy and safety of C-PDT and DL-PDT for treatment of facial flat warts in pediatric patients. They studied 30 young patients, who were divided in two group, with mean age of 9.67 ± 4.48 years (range 4–17) in group A and 9.13 ± 2.77 years (range 5–15) in group B. The two groups were randomly assigned to receive treatment with C-PDT or DL-PDT. The authors noted that in the early 12 weeks the treatment with DL-PDT seemed to fail. In fact, none of patients treated with DL-PDT reached an excellent response (75–100% reduction of total wart count), compared to 53.3% of patients treated with C-PDT. Nevertheless, this gap was filled in the following 12 weeks. At 24 weeks follow-up 80% of patients of DL-PDT group showed excellent response compared to 73.3% of patients of C-PDT group. So, in the long-term follow-up DL-PDT and C-PDT showed similar clinical efficacy for the treatment of pediatric facial flat warts. Adverse effects were also similar in the two group, with transient pain, irritation and hyperpigmentation reported (Borgia et al., 2020a). At 1-year follow-up, 60% of patients of both group (DL-PDT and C-PDT) maintained excellent response (75–100% reduction of total wart count compared with baseline). After 24 weeks, among the responders, 13.3% of C-PDT–treated patients and 20% of those treated with DL-PDT experienced mild relapses in terms of lesions’ number and size. None of non-responders at 24 weeks achieved improvement at 1-year follow-up. No long-term side effects were reported in both groups (Borgia et al., 2021).

### Genital viral warts

PDT may be particularly useful for genital warts difficult to treat due to their localization.

Shan et al. (2016) described the efficacy of PDT in the treatment of genital viral warts in male urethra. They treated 76 men including pediatric patients (mean age 32.6 years, range 16–65) applying 20% ALA solution with a thin cotton swab gently inserted into the urethra. After a 3 h incubation period, they irradiated the lesions with a urethral cylindrical semiconductor laser fiber emitting light of 635 nm wavelength. The treatment was repeated once every week for 4 weeks. At the 3 months follow-up, almost all patients had a complete response and only five (6.6%) patients relapsed. Of these, three received four more sessions of PDT resulting in clearance of lesions without further recurrence.

Overall nine pediatric patients with genital viral warts were treated with ALA-PDT, achieving complete response in all cases (Xu et al., 2018; Macca et al., 2022).

Xu et al. (2018) reported eight treatment-resistant cases of pediatric genital warts successfully treated with 20% ALA-PDT. They irradiated perianal and intra-anal areas with red light from a semiconductor laser. Pretreatment by using microwave ablation was applied for lesions larger 5 cm. The patients were sedated with oral chloral hydrate (0.5–0.8 mg/kg) half an hour before light exposure. The majority of patients achieved complete response after three to six PDT sessions, but one patient required up to 12 sessions. The patients experienced mild to moderate pain during light exposure, according to a pain score. At a 6-month follow-up, neither other side effects nor recurrences were detected.

Macca et al. (2022) proven the effectiveness of ALA-PDT on non-sexually transmitted genital warts of a 5-year-old female. They applied 10% ALA ointment and irradiated with red light after an incubation period of 3 h. At 3 months follow-up, only a few flat elements were still visible but after further 3 months complete clearance was detected. The patient experienced mild to moderate burning sensation during light exposure and a transient hyperpigmentation, with no long-term side effects at 6 months follow-up. In conclusion, PDT may be considered as first-line therapy in patients with high number of genital warts, for which other topical therapies are excessively expensive or painful, or in patients with warts in urethral or anal and perianal areas. Xu et al. (2018) proven that the rapid healing of PDT makes it an optimal therapeutic option for pediatric genital warts in perianal and intra-anal areas, where other invasive treatments may cause anal stenosis and difficult defecation. Similarly, Shan et al. (2016) successfully used PDT for urethral genital warts in patients of all ages, including children.

### Cutaneous leishmaniasis

In addition to treatment of viral and bacterial disease, PDT in pediatric age has been studied for management of parasitic infection from Leishmania. Mechanisms underlying effects of PDT on cutaneous leishmaniasis are largely unknown. Amastigotes are proven to accumulate very low amounts of protoporphyrin IX and some species of Leishmania lack enzymes of heme synthesis. Despite this, PDT may exert its effects on this type of protozoa by increasing the local temperature of the skin: hyperthermia treatment has been described as an effective therapeutic option for cutaneous leishmaniasis (Fink et al., 2016). Johansen et al. described an ulcerative resistant case of cutaneous leishmaniasis by Leishmania major in a 15-year-old boy successfully treated by 10% ALA-PDT. They performed conventional PDT twice weekly for 12 weeks. The patient had previously received unsuccessful treatments, including meglumime antimoniate, topical ketoconazole, and oral fluconazole. The authors noted full healing of the ulcer 1 month after PDT. Pain experienced during treatment was controlled by topical application of lidocaine (Johansen et al., 2019). Leishmania species that can cause mucocutaneous (*L. braziliensis* complex) or visceral leishmaniasis (*L. donovani* complex) should not be treated with PDT.

However, currently PDT is indicated only for cutaneous leishmaniasis resistant to other treatments and in aesthetically sensitive parts of the body (Morton et al., 2020).

## Miscellanea

### Vitiligo

Vitiligo is a common progressive depigmentation of the skin due to selective destruction of melanocytes (Vaccaro et al., 2015; Vaccaro et al., 2017a). Although the pathogenesis remains scarcely known, it seems to be related to genetic predisposing factors, oxidative stress and autoimmune dysregulation (Vaccaro et al., 2016; Vaccaro et al., 2017b; Custurone et al., 2021). PDT is proven to inhibit melanogenesis *in vitro*, reducing melanocytes melanin content and tyrosinase activity. *In vivo*, PDT reduces mottled hyperpigmentation of photoaged patient skin (Kim et al., 2018). These observations may partially explain why PDT did not achieve brilliant results in treatment of vitiligo in pediatric patients. Zhang et al. (2018) conducted a study to determine the effective PS concentration, PS application duration, irradiation duration, and irradiation dosage for the treatment of vitiligo. They selected ALA concentration of 1.5%, PS application duration of 3 h, irradiation duration of 20 min, and irradiation dosage of 80 mw/cm^2^ as the better parameters for treating their patients. They treated vitiligo in two pediatric patients (4 and 17 years). Nevertheless, the results were contrasting and characterized by alternating periods of worsening and improvement. In the 17-year-old patient, pigment islands around skin follicles of vitiliginous areas of abdomen increased significantly with the number of early treatments. Nevertheless, during subsequent treatments at long intervals, pigment islands decreases progressively and, at the end of follow-up, no significant changes in pigmentation were detected compared to baseline. In the 4-year-old patient, vitiligo was found on forehead. During PDT treatment, pigment islands increased significantly first near the left eyebrow, then on the forehead and finally near the hairline, with an apparent general improvement of vitiligo area compared to baseline.

When compared to topical corticosteroids, a standard treatment for vitiligo, PDT does not demonstrate any additional therapeutic effects (Rahimi et al., 2021). For that reason, there are no reasons to prefer it to other available therapies.

### Angiofibromas of tuberous sclerosis

Weinberger et al. (2009) associated ALA-PDT with pulsed dye laser (PDL) to treat angiofibromas of tuberous sclerosis of six young patients. Two of these were in pediatric age (10 and 18 years). They combined 417-nm blue light with 595-nm PDL after application of 20% ALA solution and obtained decrease of lesions number and size. Transient side effects included erythema, swelling and superficial desquamation.

### Verrucous epidermal nevus

Zheng et al. (2018) combined fractional micro-plasma radiofrequency (RF) technology and PDT to treat a facial verrucous epidermal nevus (VEN) in a 17-year-old girl. They carried out local anesthesia by using lidocaine cream under occlusion an hour before the therapy. Then, they performed the fractional micro-plasma RF treatment, which caused a temporary volume decrease of verrucous papules, and the first treatment of ALA-PDT 4 h later. Other three ALA-PDT session without RF pretreatment were performed. After treatment, the patient achieved complete disappearance of warty lesions on her face and no recurrences of VEN were detected at 24 weeks follow-up.

### Linear porokeratosis

MAL-PDT has proven to be very effective in the treatment of Linear porokeratosis (LP), is a disorder of keratinization typically occurring in pediatric age. García-Navarro et al. (2009) obtained a cosmetic and clinical improvement of a LP on lower leg of a 13-year-old boy by using 16% MAL-PDT and red light. They performed two PDT sessions 1 month apart. No recurrences were observed after 11 months.

Curkova et al. (2014) performed three MAL-PDT sessions on extensive LP of a 16-year-old girl. They noted a progressive improvement of her multiple reddish-brown macules and depressions on right arm and at 1-year follow-up the cosmetic and clinical response was considered satisfactory. They did not performed pretreatment except for removal of superficial scale before second PDT session. The patient experienced only a transient burning sensation during illumination.

Garrido-Colmenero et al. (2015) reported the case of an 11-year-old girl with LP on her left breast. They did not obtain clinical improvement by using topical therapies, including adapalene and tacrolimus 0.1% ointment so the patient underwent two sessions of MAL-PDT with good result. Four months after PDT, only few lesions remained.

Gracia-Cazaña et al. (2015) used MAL-PDT to treat porokeratosis in children with bone marrow transplant. A 12-year-old boy presented three round lesions in the right popliteal fossa and two in the cervical region, histologically diagnosed as porokeratosis. After three sessions of MAL-PDT, he achieved complete clearance and remained free of disease after 5 years follow-up.

## Discussion

Despite its worldwide use in adult patients that has provided strong evidence about efficacy and safety not only for oncologic conditions but also for inflammatory and infectious diseases, PDT applied to the pediatric population appears to be a substantially unexplored continent. Our review has in fact evidenced that this peculiar kind of photochemotherapy has been investigated in few diseases, with a very limited number of RCT and small case series, while most of our knowledge in children originates from sporadic case reports on single patients. This find an obvious justification regards to skin tumors, which occurrence in pediatric age is very rare, mainly represented by keratinocyte cancer in syndromic patients. The consequent almost complete absence of data about its effectiveness in the face of the not estimable risk of worsening or relapse strongly suggest, for ethical reason, its use only in exceptional cases. PDT could be considered therapeutic alternative in case of benign lymphocytic infiltration of the skin, while its use in cutaneous lymphomas may be hypothesized in a near future, as proposed for adult patients, only in localized form when other therapies are contraindicated or have failed (Morton et al., 2020). Moreover, as demonstrated in adults patients (Hooper et al., 2021) and suggested for children by Heng et al. and Mendese et al., repeated sessions of PDT are needed to obtain a clinical response in cutaneous lymphomas such as MF, thus potentially limiting the feasibility of this type of treatment. The anti-inflammatory effects of PDT have been studied in diseases mainly affecting the pilosebaceous unit, such as acne and HS. Both acne and HS have a dramatic burden, negatively conditioning the everyday life of the patients, especially in a delicate period such as adolescence, with disastrous effect on affective, social and sexual aspects resulting in low self-esteem feelings and depression. The point of strength of PDT in such cases seems to be its ability to hit with one shot three different pathogenic mechanisms, inhibiting the proliferation of *P. acnes*, targeting activated T lymphocytes thus reducing the release of cytokines which attract leucocytes to dermis and improving follicular hyperkeratosis acting on keratinocytes differentiation and proliferation. Examining the available data on this topic, no significant advantages emerge respect to both topical and/or systemic conventional therapies. To date, PDT may be considered as a valid second-line treatment in patients resistant to antibiotics and/or retinoids or when such therapies are contraindicated. Some types of acne, especially the nodulocystic form of the face and trunk, may largely benefit from PDT not only to achieve sustained clinical improvement but also to reduce the risk of permanent scarring. PDT is known to promote the remodeling of the dermal matrix architecture *via* keratinocyte photoactivation with subsequent paracrine induction of matrix metalloproteinases production in fibroblasts. Its use at an early stage of the disease may accelerate resolution of the cystic lesions, reducing the risk and the severity of disfiguring scars. On the basis of these consideration, PDT could find growing application also in pediatric HS patients, a highly disabling disease with an always increasing incidence in pre-puberal and puberal patients, especially in those affected by concomitant predisposing factors such as obesity. More than in acne, PDT could help to control inflammation in a non-invasive way lowering the necessity to recur to prolonged systemic therapies not free from long-term side effects (antibiotic resistance), partially contraindicated (tetracyclines) and often not well accepted by both little patients and their parents. Moreover, its use since the onset of the disease may reduce the frequency and the severity of inflammatory episodes, preventing the development of undesirable scars with both cosmetic and functional impairment. An interesting field of application of PDT in pediatric patients is undoubtedly the antimicrobial one, with particular regards to HPV infection. With respect to the aforementioned indications, there are enough experimental and clinical experiences to affirm that PDT can be considered an effective, safe and well tolerated solution for both cutaneous and mucosal warts. Conventional therapies, including topical keratolytic agents, electrosurgery, cryotherapy and carbon dioxide laser may cause scars, inflammatory reactions and hyper- or hypopigmentation, with high risk of treatment failure and recurrence. Furthermore, such treatments are often contraindicated or not tolerated, especially in children. In general, lesion-directed therapies are not fully effective to eradicate HPV infection, in particular in subclinical and latent conditions. Conventional PDT has been used in children with good cosmetic results, better compliance and lower recurrence rates. However, it shows some limits: it is accompanied by pain during illumination, is time-consuming and requires dedicated equipment. DL-PDT offers advantages over C-PDT in terms of tolerability, time and cost, making this procedure more suited to the pediatric setting. DL-PDT is a novel procedure in which the activation of the topical photosensitizer is induced by exposure to natural daylight, without requiring preliminary occlusion. The absence of occlusion, with consequent less time spent at the clinic and the possibility to perform the treatment in an outdoor setting, may increase the compliance of young patients. In addition, pain intensity during DL-PDT is significantly lower than with C-PDT, probably because of gradual and continuous production and photoactivation of smaller amounts of protoporphyrin IX, minimizing the little patient’s discomfort during irradiation.

With regards to the safety profile, as reported by all aforementioned studies, PDT is associated with only transient and mild to moderate adverse effects in pediatric population, such as those observed in the adult population. PDT side effects can be classified in early (immediately or within days after treatment) and late (after weeks or months) onset side effects. Early onset side effects include pain and local skin reactions (LSRs), namely, erythema, burning, edema, crusting, desquamation, or pustules (Borgia et al., 2018b). Pain is the more common adverse effects, but rarely requires analgesia. DL-PDT is considerably and statistically significantly less associated with pain than C-PDT. Borgia et al. observed that post-irradiation pain was similar in DL-PDT and C-PDT groups of pediatric patients, but further studies are needed to compare the two modalities and to evaluate any differences in terms of safety (Borgia et al., 2020a). Pain during PDT in pediatric patients may be also related to disease localization. Head and neck district is one of sites most associated with treatment-related pain. In their study on treatment of HS, Bu et al. proven that PDT did not requires previous analgesia except in the case of a 16-year-old boy who presented HS lesion in craniofacial and neck areas (Bu et al., 2017). Hyperpigmentation is a worrisome side effect, especially in children with high phototype skin, but may be avoided using appropriate photosensitizer concentration and incubation time and most often is transient and disappears spontaneously after a few weeks or months. Other side effects have been described following PDT, such as onychodystrophy, hair loss, erosive pustular dermatosis of the scalp and urticarial reaction urticarial reaction (Guarneri and Vaccaro 2009). Urticaria-like reaction was reported in two pediatric patients following a few minutes of light exposure. The first patient was an 11-year-old girl with Gorlin syndrome who was treated for BCC, while the second patient was a 4-year-old girl who was treated for porokeratosis. In the first patient, a subsequent provocation skin test confirmed that the reaction was produced by the combination of MAL and illumination while in the second patient, provocation testing was not carried out due to the her young age (Miguélez et al., 2013). No information regarding long-term safety of PDT in pediatric patients are available (Snider et al., 2020). The main concern is the development of PDT-induced skin cancers, as reported in some cases in adult patients (Borgia et al., 2018b). To date, there is no evidence that PDT can stimulate skin carcinogenesis in children but a continuous and careful follow-up of PDT-treated patients is needed to verify this hypothesis.

## Conclusion

Clinical trials focusing on PDT treatment in children are rare. There is a general reluctance about involving children in trials by parents and adults, especially because of fears of unpredictable side effects in the pediatric population. Moreover, trials on children involve more ethical concerns because children lack the capacity to understand the risks underlying trials and informed consent is difficult to obtain by parents (Joseph et al., 2015). Nevertheless, the review of the available data has showed promising results, with some points of strength but even a bigger number of uncertainties. It appears as a safe therapeutic procedure. Pain may limit the compliance of pediatric patients but previous local analgesia or more tolerable PDT settings, for example, daylight PDT, lower PS concentration, shorter incubation times or lower light fluences, may be useful in more sensitive patients. These parameters should be modulated in the same way to avoid the risk of hyperpigmentation, which represents a particularly worrisome side effect on a child’s face. Nevertheless, PDT-induced hyperpigmentation is generally transient and responsive to local treatment. To date, there are no reports of PDT-induced skin cancers in pediatric age, so this possible adverse events remains only theoretical. PDT may be a soft procedure in children because it does not require daily treatments but limited in number and spaced in time. Nevertheless, it is time-consuming because the patients have to wait a number of hours at the hospital between PS application and illumination. Daylight PDT may be useful to reduce waiting times and it better fits to pediatric patients who would rather spend time outside than within the walls of an hospital. Data on efficacy of DL-PDT in pediatric patients are still limited, and further comparison between C-PDT and DL-PDT is needed in this population. PDT has the advantage that it can be easily combined with other therapies, thus increasing its effectiveness rates. The majority of local combination therapies, such as curettage, microneedling, fractional micro-plasma radiofrequency, radiant infrared or surgical debulking, were used to improve penetration of photosensitizer in the skin. Instead, other therapies with mechanism of action other than PDT, such as cryotherapy for viral warts or oral antibiotics for juvenile acne, can also be associated without increased risk of side effects. Nevertheless, the analysis of the literature has evidenced a number of questions that need to be addressed relatively to the most appropriate type, concentrations, and incubation period of photosensitizers, and optimal parameters of illumination sources in the different pathologies, adapting them in the light of the clinical characteristic of each single patient including age, disease severity, extent of the disease and its localization in different areas of the body. Unfortunately, the great heterogeneity of light sources, formulations, and photosensitizer types reported in literature makes comparison and analysis difficult. Moreover, PDT has been used with satisfying results in adults for other dermatologic conditions which may be explored also in pediatric patients, including connective tissue disorders, such as chronic lupus erythematosus or morphea/scleroderma, genital and oral lichen planus, lichen sclerosus, and several types of scars (Kvaal et al., 2013; Gordon Spratt et al., 2015; Wennberg, 2015; Borgia et al., 2016b; Gerkowicz et al., 2021). Specific protocols for pediatric patients as well as the length of follow-up intervals must be better standardized in larger RCT studies in order to draw up shared guidelines taking full advantage from such versatile treatment.
